# Evolution of Specimen Self-Collection in the COVID-19 Era: Implications for Population Health Management of Infectious Disease

**DOI:** 10.1089/pop.2020.0296

**Published:** 2021-02-05

**Authors:** Franklin R. Cockerill, Jay G. Wohlgemuth, Jeff Radcliff, Christine E. Sabol, Hema Kapoor, Jeffrey S. Dlott, Elizabeth M. Marlowe, Nigel J. Clarke

**Affiliations:** ^1^ Rush University Medical Center, Chicago, Illinois, USA.; ^2^ Quest Diagnostics, Secaucus, New Jersey, USA.

**Keywords:** 2019 novel coronavirus disease (COVID-19), severe acute respiratory syndrome coronavirus 2 (SARS-CoV-2), respiratory tract infections, self-collection, specimen handling, telemedicine

## Abstract

Laboratory testing is an important component in the diagnosis of respiratory tract infections such as with severe acute respiratory syndrome coronavirus-2 (SARS-CoV-2). However, specimen collection not only risks exposure of health care workers and other patients to infection, but also necessitates use of personal protective equipment that may be in short supply during periods of heightened disease activity. Self-collection of nasal or oropharyngeal swabs offers an alternative to address these drawbacks. Although studies in the past decade have demonstrated the utility of this approach for respiratory infections, it has not been widely adopted in routine clinical practice. The rapid spread of coronavirus disease 2019 (COVID-19), caused by SARS-CoV-2, has focused attention on the need for safe, convenient, timely, and scalable methods for collecting upper respiratory specimens for testing. The goals of this article are to highlight the literature regarding self-collected nasal or oropharyngeal specimens for respiratory pathogen testing; discuss the role of self-collection in helping prevent the spread of the COVID-19 disease from infected patients and facilitating a shift toward “virtual” medicine or telemedicine; and describe the current and future state of self-collection for infectious agents, and the impacts these approaches can have on population health management and disease diagnosis and prevention.

## Introduction

Numerous studies demonstrating the utility of self-collected nasal or oropharyngeal specimens for respiratory viruses have been published over the last decade. However, adoption of this specimen collection method for upper respiratory infections has been slow and tentative. With the rapid spread of coronavirus disease 2019 (COVID-19), caused by severe acute respiratory syndrome coronavirus-2 (SARS CoV-2), development of safe, convenient, timely, and scalable methods for collecting upper respiratory specimens for testing has become paramount.

One approach to improve the safety of specimen collection is for patients to collect the specimens themselves, either at home or under supervision by a health care professional (HCP). Both approaches limit the potential for transmission of SARS-CoV-2: supervised self-collection at a health care venue limits the need for close contact between patients and HCPs, while at-home self-collection also avoids interaction among patients.

Self-collection of nasal specimens also can reduce workload for medical staff and lessen the requirement for personal protective equipment (PPE), which had been in low supply early in the pandemic. Finally, self-collection of nasal specimens facilitates diagnostic testing as part of “virtual” medicine or telemedicine, which has become an important care delivery method during the COVID-19 pandemic. Self-collection of specimens as part of telemedicine-based care can help in other areas of testing as well, by reducing unnecessary exposure during clinician office visits and allowing for continuity of screening and monitoring when in-person clinician visits are not feasible.

The intent of this article is 3-fold: (1) to briefly highlight the literature related to self-collection of nasal or oropharyngeal specimens for testing respiratory pathogens, including SARS-CoV-2; (2) to provide an example of a real-time practice algorithm for SARS-CoV-2 self-collection; and (3) to describe the current and future state of self-collection—primarily for respiratory infections—and the impacts these processes can have on population health management and disease prevention.

## Clinical Studies of Self-Collected Upper Respiratory Specimens for Respiratory Pathogen Testing

The development of nucleic acid amplification tests (NAATs) for clinical use in the early 1990s permitted the detection of infectious agents at lower concentrations in human specimens, as compared to traditional culture techniques. The enhanced sensitivity of NAATs has facilitated less invasive sampling techniques, including the use of respiratory secretions from more easily accessible sources than the nasopharynx—including the anterior nares, mid-turbinate area of the nose, and oropharynx, as well as saliva and sputum. As a result, self-collection of respiratory specimens by patients has become more feasible. Studies over the past decade have compared self-collection of respiratory specimens with collection by HCPs, most of which have focused on influenza and group A *Streptococcus* testing.

The following discussion describes clinical studies that have been reported for self-collection of specimens for frequently encountered respiratory pathogens, including SARS-CoV-2. Studies included in this review were prospective clinical evaluations, whereby a self-collected respiratory specimen was compared with an HCP health care-collected respiratory specimen and tested for respiratory pathogens in patients experiencing upper respiratory symptoms.

Search terms included the following, alone or in combination: self-swabbing, self-collection, self-collected swabs, saliva, respiratory illness, influenza, COVID-19, SARS-CoV-2, virus, group A *Streptococcus*, respiratory pathogens, influenza A, influenza B, respiratory syncytial virus (RSV), parainfluenza virus, human coronavirus (hCoV), adenovirus. Studies also were identified from the bibliographies of published work on the same topic. Once identified, some studies were not included in the review because of low numbers of cases, lack of adequate comparators, lack of peer review, the use of small or targeted populations, and lack of direct comparisons between collection methods.

Table 1 provides a summary of studies discussed in the following sections that include information on agreement of results between self-collected and HCP-collected specimens.

**Table 1.** Summary of Clinical Studies Assessing Agreement of Self-Collected Versus Health Care Professional–Collected Nasal or Oropharyngeal Specimens for Respiratory Pathogen Testing^a^

Study	Study size, paired specimens	Age range	Self-collected specimen	Findings
**Influenza A, B**				
Dhiman, et al^1^	58	18–92 y	MT	**Agreement**: 94.8% (55/58)
				**Positivity rate^b^:** 34.4% (20/58)
Esposito, et al^2^	203	6 mo-5 y	MT	**Agreement:** 96.5% (196/203)
				**Positivity rate**: 15.8% (32/203)
**Multiple upper respiratory viruses**				
Akmatov, et al^9^	75	28–46 y	AN	**Agreement:** 93.3% (70/75)
				**Positivity rate**: 36% (27/75)
Larios, et al^8^	76	23–59 y	MT	**Agreement**: 90.8% (69/76)
				**Positivity rate**: 38.2% (29/76)
**SARS-CoV-2**				
Tu, et al^11^	530	15 mo-94 y	Tongue, nasal, MT	**Agreement:**
				Tongue: 98.6% (494/501)
				Nasal: 98.2% (494/498)
				MT: 99.6% (502/504)
				**Positivity with self or HCP-collected swab:**
				Tongue: 10.2% (51/501)
				Nasal: 10.2% (51/498)
				MT: 10.3% (52/504)
Wehrhahn et al^12^	236	9–81 y	Nasal or throat	**Agreement:** 99.6% (235/236)^c^
				**Positivity rate:** 10.6% (25/236)
Therchilsen et al^46^	109	>18 y	MT or OP	**Agreement:** 95.4% (104/109)
				**Positivity rate:** 17.4% (19/109)
				
				
Cheuk et al^20^	229	17–36 y	POS	**Agreement: 75.9% (174/229)**
				**Positivity rate: 69.4% (159/229)**
Landry et al^22^	124	Not available	Saliva	**Agreement: 94% (117/124)**
				**Positivity rate: 26.6% (35/124)**
Leung et al^21^	95	19–85 y	DTS	**Agreement: 78.9% (75/95)**
				**Positivity by NP: 47.4% (45/95)**
Jamal et al^47^	91	23–106 y	Saliva	**Agreement: 69.2% (63/91)**
				**Positivity rate by NP or Saliva: 79.1% (72/91)_**
Wyllie et al^23^	70 inpatients		Saliva	70 patients with positive NP by HCP at hospital admission. Follow-up comparison of self-collected saliva vs. HCP NP swab. Higher SARS-CoV-2 copy number in saliva (mean log copies/mL 5.58; 95% CI 5.09–6.07) than in NP collected by HCP at the same time (mean log copies/mL 4.93; 95% CI 4.53–5.33)
**Group A Streptococcus (*Streptococcus pyogenes*)**				
Murray, et al^26^	363	4–72 y	OP	**Agreement**: 93.7% (340/363)
				**Positivity rate**: 37.7% (137/363)

^a^ Agreement refers to agreement of results from the self-collected specimen with those obtained with the HCP-collected specimen.

^b^ Positivity rate determined as proportion with positive results using at least 1 of the sample collection methods.

^c^ Calculated from Table 1 of reference 12, which showed detection of SARS-CoV-2 by either method for 25/236 patients (by self-collection in all 25 and by HCP collection in 24). Other respiratory viruses included in the analysis were not considered in this agreement rate.

Parents or guardians collected specimens for young children.

AN, anterior nares; DTS, deep throat saliva; HCP, health care professional; MT, mid-turbinate; NP, nasopharyngeal; OP, oropharyngeal; POS, posterior oropharyngeal saliva; SARS-CoV-2, severe acute respiratory syndrome coronavirus-2; y, years.

### Influenza A and B

Several studies have documented the utility of self-collected specimens for detection of influenza infection. Nearly a decade ago, Dhiman and colleagues conducted a study of 58 adults (ages 18–92 years) with flu-like symptoms and no prior health care training.^1^ Swabs were collected from the mid-turbinate area of the nose by the patients, and separately by an HCP, and tested using a real-time reverse transcription-polymerase chain reaction (RT-PCR) method. Of the 58 paired specimens tested, 20 were positive for influenza A or B viruses by a least 1 collection method, representing an overall positivity rate of 34.4%. Agreement between patient-collected and HCP-collected nasal swabs for detection of influenza A or B viruses was 94.8% (Table 1); results were positive only by self-collection for 2 (3.4%) of the 58 swabs and only by HCP collection for 1 (1.7%). In addition to good test performance, more than half (53.4%) of patients preferred self-collection over HCP collection, and many (25.9%) had no preference.

Specimen “self”-collection also appears to be feasible for children. Esposito and colleagues evaluated 203 young children (ages 6 months to 5 years) with respiratory disease symptoms for whom specimens were collected for detection of influenza A and B viruses by RT-PCR.^2^ Mid-turbinate nasal swabs were collected by parents or guardians of the children, as well as by pediatricians. Agreement between parent-collected and HCP-collected specimens was 96.5% (196 of 203 paired specimens). Thirty-two specimens were positive by at least 1 collection method, representing an overall positivity rate of 15.8%; results were positive only by self-collection in 4 (2%) of the 203 infants, and only by pediatrician collection in 3 (1.5%). Importantly, children were more satisfied with parental collection than with pediatrician collection, which could facilitate specimen collection.

A recent meta-analysis evaluated several self-collection studies for influenza (including the aforementioned Dhiman and Esposito studies), with the assumption that bias could be introduced if patients self-reported symptoms and then self-collected on their own.^3^ In other words, if patients do not collect samples at appropriate times, community prevalence data derived from self-collection would be lower than if derived from HCP evaluation and testing. Despite this potential bias, patient self-collection showed a pooled sensitivity of 87% and pooled specificity of 99% relative to collection by HCPs.^3^ Other studies have reported favorable results for self-collection versus HCP-collected specimens for influenza A and B viruses but are not included here because of low numbers of cases, lack of adequate comparators, or the use of small, targeted populations.^4–7^

### Other common respiratory viruses, including coronaviruses

Fewer studies exist for self-swabbing and detection of viruses beyond influenza viruses. Larios and colleagues^8^ compared mid-turbinate swabs self-collected by patients with nasopharyngeal swabs collected by nurses for influenza A and B; RSV A and B; parainfluenza virus 1, 2, and 3; hCoV 229/NL63; hCoV OC43/HKU1; rhinovirus A/B, adenovirus; and human metapneumovirus. Detection used a multiplex PCR testing method, and a total of 38 patients (age range 18–69 years) with 76 episodes of respiratory illness were evaluated (Table 1). Results agreed in 90.8% of episodes (69 of 76 paired specimens). Twenty-nine specimens were positive by at least 1 collection method, representing an overall positivity rate of 38%; virus was detected with mid-turbinate self-collection alone in 4 (5%) episodes and with nasopharyngeal nurse collection alone in 3 (4%). Relevant to the current COVID-19 pandemic, self-collection was demonstrated to allow adequate detection of coronaviruses: coronaviruses were detected in equal frequency by mid-turbinate self-collection (n = 20) and nasopharyngeal nurse collection (n = 20).

Akmatov and colleagues also compared self-collected anterior nares swabs with HCP-collected anterior nares swabs for RT-PCR detection of influenza A and B; rhinovirus A/B/C; hCoV 229E/NL63 and OC43; parainfluenza virus 1, 2, 3, and 4; RSV A and B; adenovirus A/B/C/D/E; human metapneumovirus; enterovirus; and bocavirus 1/2/3/4.^9^ Agreement between the 2 swab types was high, at 93.3% (70 of 75 paired specimens). Twenty-seven specimens were positive by at least 1 collection method, representing an overall positivity rate of 36%; in 4 cases infection was detected with self-collection versus 1 case that was only detected with HCP collection (Table 1). In a recent phase-2 RSV vaccine trial, self-collected nasal swabs compared well with site-collected nasal swabs for detection of RSV.^10^

### SARS-CoV-2: etiologic agent of COVID-19 disease

Several studies to date have looked at the performance of self-collected specimens for detection of SARS-CoV-2, and results have been encouraging. An analysis by Tu and colleagues compared SARS-CoV-2 detection using a variety of swab types self-collected (under HCP observation) by 530 symptomatic child and adult outpatients (or their parents or guardians), with nasopharyngeal swabs collected by HCPs as the comparator gold standard.^11^ Of the nasopharyngeal swabs collected by the HCP, 51 (9.6%) were positive. Self-collected specimens showed relatively high sensitivity, although the lower bound of the 1-way 97.5% confidence interval (CI) included 90% for each; estimated sensitivities were 89.8% (97.5% CI, 78.2% to 100%) for tongue, 94.0% (83.8% to 100%) for anterior nasal, and 96.2% (87.0% to 100%) for mid-turbinate swab specimens.

Similar results were reported in a study of children and adults in Australia: detection of SARS-CoV-2 was similar in self-collected nasal and throat swabs (25/236) as in paired HCP-collected specimens (24/236), as was detection of other respiratory viruses (58 detected with self-collected specimens, 56 with HCP-collected specimens).^12^

In a study by McCulloch and colleagues, lower sensitivity of self-collection was observed; however, self-collection was performed an average of 1 day later than the comparative HCP collection.^13^ In this cross-sectional study enrolling 185 symptomatic adults (158 presenting at a drive-through testing site and 27 enrolled after a positive result for SARS-CoV-2), mid-nasal swabs collected by patients yielded only 80% (95% CI, 63%-91%) sensitivity and 97.9% (95% CI, 94%-99.5%) specificity relative to testing of nasopharyngeal swabs collected by a clinician.^13^ Viral load appeared to play a role: among patients with a relatively high viral load (cycle threshold ≤32) on HCP-collected specimens, self-collected specimens yielded 95% sensitivity.^13^ As mentioned, a major limitation of this study was that at-home collection was performed on average a day after HCP collection; this has the potential to decrease detection rates, given that viral load tends to decline over time. This limitation is not uncommon in studies of self-collection for SARS-CoV-2 testing.

In addition to nasal and throat specimens, self-collected sputum and saliva also have been studied for detection of SARS-CoV-2. For example, COVID-19 patients with pneumonia tend to have high viral loads, and self-collected sputum has been suggested as a preferred specimen type for diagnosis and follow-up of these patients.^14^ In a small study of 26 specimens from 3 COVID-19 patients with pneumonia, sputum yielded higher SARS-CoV-2 ribonucleic acid (RNA) concentration over a longer time compared to nasopharyngeal swabs.^14^

Saliva also has been reported to be a useful and reliable specimen type for SARS-CoV-2 testing, with peak viral loads early during infection.^15^ An early systematic review looked at the reliability of saliva as a specimen type for diagnostic SARS-CoV-2 RT-PCR testing in hospitalized patients with confirmed COVID-19.^16^ The reported detection rates from studies with at least 10 patients ranged from about 84% to 100%,^15,17–19^ and most studies did not find significant differences in viral load between saliva and nasopharyngeal swabs.^16^ Subsequent studies have shown agreement of approximately 76% to 94% between posterior oropharyngeal saliva and nasopharyngeal swabs (Table 1).^20–22^ A relatively large study of 239 patients with 2130 self-collected posterior oropharyngeal saliva specimens and 8438 HCP-collected nasopharyngeal swabs showed somewhat higher positivity with saliva (61.5%, 95% CI 55.1%–67.6%) than nasopharyngeal swabs (53.3%, 95% CI 46.8%–59.6%).^20^ The positive percent agreement between saliva and nasopharyngeal swabs was higher when samples were obtained within 7 days after symptom onset (96.6%, 95% CI 87.3%–99.4%) rather than later (75.0%, 95% CI 61.4%–85.2%).

Landry et al also found good agreement (94%) between self-collected saliva specimens and residual nasopharyngeal specimens in a series of 124 symptomatic outpatients with suspected COVID-19.^22^ Saliva yielded somewhat lower sensitivity (85.7%; 95% CI 70.6%–93.7%) than did nasopharyngeal swabs specimens (94.3%; 95% CI 81.4%–99.0%), and higher cycle threshold values (implying lower viral load). More recently, in a study of 70 inpatients with confirmed COVID-19, Wyllie and colleagues reported a higher mean viral load in saliva than in nasopharyngeal swab specimens collected at the same time by HCPs.^23^ Based on evaluation of serial specimens, sensitivity of saliva was at least similar to that of nasopharyngeal specimens.^23^

Despite the potential of saliva for self-collection, a prospective analysis among mostly symptomatic patients found RT-PCR positivity rates to be lower with deep-throat saliva (DTS; 69%) than with other respiratory specimens, including self-collected cough-out sputum (89%) and pooled nasal and throat swabs collected by health care workers (81%).^24^ As already noted, several different types of saliva sampling techniques have been used in COVID-19 studies, including simple drooling (patient allows saliva to flow directly into a collection container); posterior oropharyngeal saliva collection (specimens are obtained by coughing up and clearing the throat); DTS (obtained early morning before brushing teeth, after gargling with patient's own saliva); and spitting 1 to 2 mL into a container with or without first allowing saliva to pool in the mouth.

Although studies using the various methods have all shown the promise of saliva as a useful and convenient specimen type for self-collection, each has potential drawbacks. For example, spitting a certain volume of saliva could lead to dilute specimens with decreased viral load, while posterior oropharyngeal saliva from symptomatic patients may tend to be thick and more difficult to pipet.^22^ Differences in saliva collection have been suggested as a source of variability among studies.^25^

### *Streptococcus pyogenes* (group A *Streptococcus*): etiologic agent of strep throat

Group A *Streptococcus* (aka *Streptococcus pyogenes*) is the most frequent cause of bacterial pharyngitis in both children and adults. Murray and colleagues demonstrated equal performance of self-collected and HCP-collected pharyngeal swabs for group A *Streptococcus* using a PCR-based testing method.^26^ For self-collection, participants (and parents) were provided written and visual instructions and a mirror, but no other guidance from an HCP. Paired specimens from 402 patients (age range 4–72 years) were evaluated; parents or guardians performed testing in children.

The overall concordance between self-collected and HCP-collected swabs was 93.7% (340 of 363 pared specimens; 95% CI, 91.3 to 96.0) (Table 1). A total of 137 specimens were positive by at least 1 collection method, representing an overall positivity rate of 37.7%. Of the 24 paired swabs with discordant results, 10 were detected only with HCPs swabbing and 14 only by self-swabbing (*P* = 0.41). Of the 206 participants who collected their own swab, 122 graded the swab collection as very easy, 77 as easy, and 4 as difficult; 3 did not provide a response.

### Summary

As described in the foregoing sections and summarized in Table 1, numerous studies have demonstrated that self-collected respiratory specimens, including mid-turbinate, anterior nasal, and oropharyngeal, can be used for many common upper respiratory pathogens, with high levels of agreement with HCP-collected specimens. Additionally, most patients surveyed after self-swabbing reported being comfortable with performing these self-collections and would do so again when required; children preferred having their parent swab for them versus a pediatrician. Availability of at-home self-collection has the potential to increase participation in diagnostic testing as well as participation in clinical studies.

## Testing Algorithm for SARS-CoV-2 Self-Collection of Mid-Turbinate or Anterior Nasal Specimens Developed by Quest Diagnostics

Data from the study by Tu et al^11^ were useful in developing an algorithm for self-collection for SARS-CoV-2 testing that has received emergency use authorization. This represents the first “non-observed” (ie, an HCP is not required to observe the collection by the patient) collection approval for SARS-CoV-2.

With this algorithm, a physician provides pre-authorization for testing, frequently through an employee health service, telemedicine service, or traditional brick-and-mortar health care facility. A SARS-CoV-2 RT-PCR test kit is then shipped by rapid courier to the patient, often to their home address, within 1 business day. Alternatively, kits are available and distributed to patients at their worksites, community health care sites, or a patient service center in a retail or health care environment. The patient performs the self-collection and returns the secured specimen to Quest Diagnostics for testing. The entire process typically takes 3 to 4 days. The physician who ordered the test then reviews the results with the patient and takes any appropriate actions required, including observation at home or admission to the hospital.

This at-home self-collection process has advantages beyond providing test results in a relatively short period of time: patients do not have to leave their homes, the procedure does not require medical staff and their associated PPE requirements (labor and equipment costs), and it reduces opportunities for exposure of HCPs and other patients to SARS-CoV-2 in a health care environment. At-home self-collection may be particularly useful for individuals who are asymptomatic but for whom RT-PCR screens may be recommended or required; for example, for patients undergoing time-sensitive surgery or aerosol-generating medical procedures,^27^ individuals traveling to regions that require a negative SARS-CoV-2 RNA RT-PCR result, and students and employees whose institutions request screening before returning to school or work. It also may have potential for employers, including health care institutions and educational institutions, for “syndromic” testing—that is, testing of individuals who develop respiratory symptoms but can be managed at home.

An important limitation of home collection is the turnaround time, which can be 3–4 days. Turnaround time can be reduced significantly once patients can procure testing kits from a patient service center or pharmacy on demand, and/or self-testing is performed as will be described in the following section. Finally, it should be emphasized that, as in the aforementioned testing algorithm, a qualified health care provider should be involved in this process for prescriptive authority, as required, and provision of consultation and treatment plans.

## Current and Future State of Self-Collection and Self-Testing for Infectious Agents

In 2012, the Food and Drug Administration (FDA) approved the first self-collected and self-performed test for an infectious disease. This test, developed by OraSure Technologies, detects HIV antibodies from saliva. In 2017, Scanwell Health was the first company to receive FDA clearance for a smartphone application for use in the diagnosis of infections in urine. For this urinary tract infection (UTI) test, patients use a kit with the Scanwell application on their smartphone for step-by-step instructions to self-collect a mid-stream urine sample with a testing strip that includes analytes associated with UTIs (leukocytes and nitrites). After 2 minutes, patients take a photo of the test strip on the scan card. Similar to a clinical urine analyzer, the application uses computer vision algorithms to provide test results to the patient immediately. Patients then have the option to complete a telemedicine visit, using their smartphone, for antibiotic treatment from a licensed clinician. This approach of at-home, smartphone-enabled testing has the potential to detect antigens or antibodies from infectious agents using lateral flow test strips.

Self-collection of specimens also has potential for screening and diagnosis of sexually transmitted infections (STIs). At present, at-home collection of urogenital specimens with mailing to a clinical laboratory for testing has not been cleared by the FDA. However, there are FDA-approved *Chlamydia trachomatis*/*Neisseria gonnorrhea* NAAT assays for vaginal specimens self-collected in clinical settings, and specimen self-collection is also being explored for mail-in testing.^28^ Given the need for social distancing in the era of COVID-19, the National Coalition of STD Directors noted that “home-based testing and consultation for STI care and conditions makes absolute sense.”^29^ For females, self-collection of vaginal swabs for STI testing has been reported to be valid and acceptable for a number of STIs, including *Neisseria gonorrhoeae*, *Chlamydia trachomatis*, *Trichomonas vaginalis,* and *Mycoplasma genitalium,* as well as for agents of vaginitis/vaginosis.^30–33^ A recent meta-analysis found that programs offering self-collection of swab specimens for STI testing (chlamydia, gonorrhea, or trichomonas) significantly increased rates of participation in STI testing (relative risk [RR] 2.94; 95% CI 1.19 to 7.28) and detection of infection (RR 2.17; 95% CI 1.04 to 4.50).^34^ Urine is also an acceptable specimen for some NAATs for *C trachomatis* and *N gonorrhea*. However, self-collected vaginal swabs have been reported to yield greater sensitivity than first-catch urine for chlamydia and gonorrhea.^35^

For males, urine samples are used routinely for *N gonorrhoeae* and *C trachomatis* testing. Self-collection of genital and extragenital swabs also has been reported to be highly sensitive for detection of multiple STIs and is acceptable to most patients.^36^

Table 2 provides a futuristic view of potential new self-collection and self-testing methods for infectious disease pathogens. Lateral flow immunoassays to detect antibodies have gained popularity with the recent COVID-19 disease outbreak. Although many of these testing methods remain in development at the time of this writing, as already mentioned, approaches such as those by Scanwell may make it possible for patients to both self-collect and self-test using a kit and a smartphone application.

**Table 2.** Future Examples of Self-Collection and/or Self-Testing Methods for Infectious Disease Pathogens

Test	Self-collection method	Test method amenable to self-testing	Notes
COVID-19 (SARS-CoV-2) Antibody	Blood finger prick	Yes	Lateral flow assay permits direct visualization or image capture by Smartphone
COVID-19 (SARS-CoV-2) Antigen	Mid-turbinate or anterior nares swabs	Yes	Lateral flow assay permits direct visualization or image capture by Smartphone
Influenza A, B Antigen	Mid-turbinate or anterior nares swabs	Yes	Lateral flow assay permits direct visualization or image capture by Smartphone
GAS Antigen	Pharyngeal swab	Yes	Lateral flow assay permits direct visualization or image capture by Smartphone
Influenza A, B NAAT	Mid-turbinate or anterior nares swabs		Specimen sent to lab for testing
RSV NAAT	Anterior nares swabs		Specimen sent to lab for testing
GAS NAAT	Pharyngeal swab		Specimen sent to lab for testing
Enteric Pathogen NAAT/*H pylori* Antigen	Stool		Specimen sent to lab for testing
STI NAAT	Vaginal swabs or urine		Specimen sent to lab for testing
Vaginitis/vaginosis NAAT	Vaginal swabs		Specimen sent to lab for testing

COVID-19, coronavirus disease 2019; GAS, group A *Streptococcus* (*Streptococcus pyogenes*); NAAT, nucleic acid amplification test; RSV, respiratory syncytial virus; SARS-CoV-2, severe acute respiratory syndrome coronavirus-2; STI, sexually transmitted infection.

Blood spots obtained through a capillary puncture (finger prick) by patients can be used to determine antibodies for SARS-CoV-2; mid-turbinate or anterior nasal self-swabbing samples can be used for antigen testing or NAAT for respiratory viruses, such as SARS-CoV-2 and influenza A and B, and group A *Streptococcus*; and self-collected stool specimens can be tested for enteric pathogens using NAAT testing (PCR).

A nasal swab is an acceptable specimen for detecting RSV in children when a NAAT method is used, as already noted. Therefore, it is conceivable that “self-swabbing” by a parent or guardian might be useful for detecting RSV in children. Although detection of RSV is possible by antigen testing, this method has the highest sensitivity with nasopharyngeal aspirates or washes—procedures not amenable to self-collection.^37^

NAAT detection of enteric infections, including *Salmonella* spp, Shiga toxin-producing *E coli*, *Shigella* spp, *Campylobacter* group, *Vibrio* group, *Yersinia enterocolitica*, Norovirus, Rotavirus, and *Giardia lamblia*, also should be feasible from self-collected stool specimens.^38^ *Helicobacter pylori*, the bacterial agent of gastric and duodenal ulcers, also can be detected by antigen testing using stool specimens.^39^

## Discussion

The advantages of specimen self-collection and, when possible, self-testing are becoming increasingly evident. Of note, the current COVID-19 pandemic has illustrated the utility of, and need for, self-collection for diagnosis of COVID-19, an acute infectious disease that is highly contagious and infects large numbers of individuals in short periods of time. Self-collection of respiratory secretions increases the access of potentially infected individuals to testing and limits the potential for spread of COVID-19 to HCPs or other patients. Additionally, self-collection reduces the need for PPE by health care personnel, as the patients can collect their own specimens in their own homes. As seen in the literature reviewed, self-collected upper respiratory specimens yield good agreement with HCP-collected specimens, often with greater patient satisfaction and convenience.

An added advantage of self-collecting specimens at home is the potential to lower barriers to participating in SARS-CoV-2 clinical studies. A survey of more than 1400 adults representing a broad range of sociodemographic groups found a strong preference for research studies that allowed home-based self-collection.^40^ Most respondents reported that they would be willing to provide saliva (88%) or throat swab (83%) specimens for research studies if they could be self-collected at home, while fewer would be willing to provide throat swab specimens at drive-through (64%) or clinical (53%) settings. Overall, 69% of respondents reported being more inclined to participate in a study if they were able to collect the specimen at home. The same research group reported similar findings for willingness to seek diagnostic testing.^41^ Among the more than 1400 survey respondents, more were willing to be tested with a home saliva specimen (92%) or self-collected nasal swab (88%) than with swab collection at a drive-through site (71%) or clinic (60%). Similar preferences were reported for follow-up testing.^41^

Self-collection is important for other acute infections as well, including streptococcal sore throat, influenza infection, and RSV infection. Agreement of self-collected and HCP-collected specimens also remains high for these infections (Table 1). As with COVID-19, patients who can be managed at home (ie, self-limited illness not requiring hospitalization) can avoid unnecessary utilization of health care services such as emergency rooms or urgent care clinics, thereby minimizing the exposure of other patients to respiratory illness.

UTI, another acute infectious disease for which self-collection and self-testing is available, can now be managed entirely at home. With this approach, self-collection of specimens can be used in combination with telemedicine to reduce the need for in-person physician visits as well as the burden on emergency rooms and acute care clinics. As a result, better service is provided for patients at their convenience. Finally, diarrhea, if not severe and not requiring intravenous hydration, could be managed at home with the same specimen self-collection and telehealth approach and offer the same benefits for patients and providers.

Home collection of specimens and telehealth management of test interpretation and treatment for STIs is appealing for several reasons. This approach provides easy, timely access to testing and the avoidance of a potentially embarrassing visit to a clinic or personal physician.

Specimen quality for self-collection may be enhanced by providing carefully written instructions and/or easy-to-follow visual diagrams, online video demonstrations, and, in the case of telehealth, real-time video guidance via telemedicine providers (ie, “direct” observation). Direct observation also may ensure that the correct person is self-testing. The turnaround time for results can be shortened if patients are able to acquire collection kits at a local pharmacy or patient service center rather than rely on courier services. With the COVID-19 pandemic, patients have been able to self-swab or have an HCP-performed swab while sitting in their car at drive-through collection facilities.

Efforts to “flatten the curve” of SARS-CoV-2 transmission in the United States may have the unintended consequence of delaying laboratory testing and diagnoses of noninfectious conditions as well. For example, Fragala and colleagues documented decreased HbA_1c_ monitoring in patients with diabetes,^42^ and potential delays in cancer diagnoses have also been noted.^43^ Along these lines, Shaukat and Church recently emphasized the need for flexibility in screening for colorectal cancer, including the use of at-home specimen collection (ie, with fecal immunochemical testing [FIT]) combined with telemedicine.^44^

Although not covered in this review, a wide range of noninfectious chronic disorders and health-related conditions (eg, diabetes, chronic renal failure, fertility, hereditary disease, pharmacogenetics, toxin exposure) can now be managed with the required diagnostic and follow-up testing via self-collection of specimens and, increasingly, self-testing. Integration of self-collection and self-testing into virtual health care delivery systems is no longer the future. By causing temporary closure of brick-and-mortar clinics, the COVID-19 pandemic has driven adoption of both self-collection and telemedicine services—not only for acute health issues, but for chronic diseases and wellness as well. Although regulatory hurdles remain, the current pandemic environment has demonstrated that alternative health care delivery strategies are vital and potentially viable options to improve the continuum of care.

The term “population health” has been defined as “the health outcomes of a group of individuals including the distribution of such outcomes throughout this group.”^45^ Health outcomes depend to some extent on high-quality, easily accessible, safe, rapid, and affordable clinical laboratory testing. Self-collection, and in some cases self-testing, permits unprecedented fulfillment of these criteria, especially demonstrated during the COVID-19 pandemic. Self-collection and self-testing not only benefit individual patients but also have relevance for management of wellness and acute and chronic disease for various patient populations, whether they are covered under employer, private, or government health plans. Health care providers also should benefit from more timely and efficient service for patients and, in the instance of communicable infectious disease, less exposure to infected patients.

## Conclusions

In summary, the COVID-19 pandemic has created a burning platform to accelerate progress in self-collection and, more broadly, in consumer-centric home- and community-based care. This approach is critical during the pandemic. Moreover, it also has merit for other areas of health care delivery, based on increasing engagement and compliance with health care and enabling more efficient health care delivery for populations. In parallel with the rapid expansion of novel virtual patient care services, the rapid development and deployment of innovative processes for self-collection and, in some cases, self-testing should enable a more convenient, efficient, safe, and potentially cost-effective health care delivery system for the diagnosis and treatment of acute disease. Such processes also may facilitate the establishment and maintenance of wellness (preventive medicine) programs. Urgent, ongoing work is needed to deliver self-collection solutions for respiratory viruses; expanding such approaches to other areas of prevention and management of chronic disease can bring significant value to our health care system.
